# Omega-3 intake in patients with coronary artery disease: focus on recent studies

**Published:** 2010-02

**Authors:** J Aalbers

## Introduction

Despite the recommendations of the American Heart Association (AHA) and other expert groups on the value of added intake of omega-3 polyunsaturated fatty acids (ω-3 PUFA) in the secondary prevention of CAD, either from the diet (oily fish – herring, mackerel, sardines) or from fish-oil supplements which contain eicosapentaenoic acid (EPA) and docosahexaenoic acid (DHA), these safe lifestyle or medication therapies are still under-prescribed.

A recent update of the omega-3 GISSI – Prevenzione trial has continued to show beneficial reductions in major clinical events at 3.5-years’ follow up.^1^ In this 1999 placebo-controlled trial, more than 11 000 post-MI patients were randomised to receive 1 g/day of omega-3 PUFAs, vitamin E, both, or neither, on top of standard medical therapy and lifestylemodification ‘advice’, for three and a half years. The omega-3s on their own were associated with a significant 15% drop in the primary endpoint of death, nonfatal MI and stroke, a 20% decrease in all-cause mortality, and a 30% decline in cardiovascular mortality driven by a steep reduction in sudden cardiac death.

Interestingly, a recent Danish study2 of 57 053 middle-aged men and women who were healthy and cancer free has shown a lower risk of acute coronary syndromes in the men with a high fatty fish intake over a mean follow-up period of 7.6 years. A total of 1 122 cases of acute coronary syndrome (ACS) were verified during a mean follow-up period of 7.6 years. Among men, intake of fatty fish was associated with a lower risk of ACS. For men in the highest quintile of fish intake compared with the lowest quintile, the hazard ratio was 0.67 (95% CI: 0.53–0.85). The inverse association was observed for intakes above 6 g of fatty fish per day, with no obvious additional benefit observed for higher intakes. Intake of lean fish was not associated with ACS. In the women, there were fewer cases of ACS and results were not consistent.

The beneficial effect of ω-3 PUFA on reducing sudden cardiac death and acute coronary syndromes is likely to originate from their anti-thrombotic and anti-arrhymic effects. Also a stabilising effect on human atherosclerotic plaque has been reported.^3^

## Omega-3 intake in heart failure patients

A recent report^4^ from the Heart Failure Society of America 2009 Scientific Meeting points out that there are very few beneficial therapies that you can add to beta-blockers, ACE inhibitors, statins and spironolactone and actually reduce cardiovascular death by 10% in heart failure. Omega-3s fit this bill and provide this additional benefit safely.

The most solid randomised-trial evidence for such a role comes from the recent Gruppo Italiano per lo Studio della Sopravvivenza nell’Infarto Miocardico Heart Failure (GISSI-HF) trial,^5^ in which nearly 7 000 patients with chronic NYHA class II to IV heart failure received either omega-3 PUFAs from fish oil at 1 g/day or placebo.

As reported by Heartwire^4^ when the study results were announced last year, the group getting omega-3s showed a 9% drop in all-cause mortality and an 8% decline in the composite of death or cardiovascular hospitalisation over a mean of about four years. Both co-primary endpoint outcomes were significant. In a separate randomisation of the patients to either 10-mg rosuvastatin (Crestor, Astrazeneca) or placebo, the statin had no significant effect on either endpoint.^6^

An exceptionally large proportion of GISSI-HF patients had been on ACE inhibitors or angiotensin-receptor blockers, beta-blocker, and spironolactone, and still there was a significant omega-3 effect. ‘It’s tough to get incremental benefit for hard endpoints in maximally treated patients’, observed Dr Dariush Mozaffarian, Harvard University, Boston, USA.

He pointed out that for the pre-specified secondary endpoint of cardiovascular death, the difference associated with omega-3 therapy reached 10% (p = 0.045). That’s a very profound benefit’, Mozaffarian said, adding that it’s similar to the cardiovascular death reduction seen with implantable defibrillator therapy.

Further insights on the value of omega-3s may be in the offing from an upcoming primary-prevention trial that will randomise a planned 20 000 people to receive vitamin D supplements, or neither (double placebo).^7^ The Vitamin D and Omega-3 Trial (VITAL) will enroll women older than 65 and men older than 60 years and follow them for five years, starting in early 2010. Plans are for at least 25% of the population to be African- American, according to the study’s co-chair Dr Jo-Ann E Manson, Brigham and Womens Hospital, Boston, USA.

## Sources of Omega-3 PUFA

The rapid depletion of fish stocks is also driving the search for land-based sources of EPA and DHA, such as oilseed crops that are genetically engineered to produce ‘marine’-equivalent omega-3 PUFAs. Sustainable sources of omega-3 fatty acids will need to be identified if long-term cardiovascular risk reduction is to be achieved at the population level.

## References

[1] Marchioli R (2002). Circulation.

[2] Bjerregaard LJ, Joensen AM, Dethlefsen C, Jensen MK, Joensen SP (2010). Euro Heart J.

[3] Thies R, Carry JMC, Yaqoob P, Rerkasem K, Williams J, Searman CP (2003). Lancet.

[4] (2009). Heartwire.

[5] Tavazzi L, Maggioni AP, Marchioli R (2008). Lancet.

[6] Tavazzi L, Maggioni AP, Marchioli R (2008). Lancet.

[7] (2009).

